# Recent Advances in CRISPR/Cas-Based Biosensors for Protein Detection

**DOI:** 10.3390/bioengineering9100512

**Published:** 2022-09-28

**Authors:** Jing Wang, Xifang Yang, Xueliang Wang, Wanhe Wang

**Affiliations:** 1 Collaborative Innovation Center of NPU, Shanghai 201100, China; 2 Institute of Medical Research, Northwestern Polytechnical University, 127 West Youyi Road, Xi’an 710072, China; 3 Research & Development Institute of Northwestern Polytechnical University in Shenzhen, 45 South Gaoxin Road, Shenzhen 518057, China; 4 Northwestern Polytechnical University Chongqing Technology Innovation Center, Chongqing 400000, China

**Keywords:** CRISPR, protein, detection, biosensors

## Abstract

CRISPR is an acquired immune system found in prokaryotes that can accurately recognize and cleave foreign nucleic acids, and has been widely explored for gene editing and biosensing. In the past, CRISPR/Cas-based biosensors were mainly applied to detect nucleic acids in the field of biosensing, and their applications for the detection of other types of analytes were usually overlooked such as small molecules and disease-related proteins. The recent work shows that CRISPR/Cas biosensors not only provide a new tool for protein analysis, but also improve the sensitivity and specificity of protein detections. However, it lacks the latest review to summarize CRISPR/Cas-based biosensors for protein detection and elucidate their mechanisms of action, hindering the development of superior biosensors for proteins. In this review, we summarized CRISPR/Cas-based biosensors for protein detection based on their mechanism of action in three aspects: antibody-assisted CRISPR/Cas-based protein detection, aptamer-assisted CRISPR/Cas-based protein detection, and miscellaneous CRISPR/Cas-based methods for protein detection, respectively. Moreover, the prospects and challenges for CRISPR/Cas-based biosensors for protein detection are also discussed.

## 1. Introduction

Protein is an important type of disease biomarker for the early diagnosis of diseases, monitoring treatment process and outcome, and assessing prognosis [1]. Various protein detection methods have been established including immunoassay [2], biological mass spectrometry [3,4], fluorescence spectrometry [5], and electrical and electrochemical methods [6]. In particular, the immunoassays based on enzyme-linked immunosorbent assay (ELISA) [2,7] and chemiluminescence immunoassay (CLIA) [8] are currently the most commonly used protein assays, in which ELISA serves as the gold standard for protein detection in the fields of clinical diagnosis and biosafety [9]. However, the levels of protein biomarkers in clinical samples are generally very low, while a large amount of matrix interference exists in samples [10], so ELISA assays in clinical use are generally limited by their sensitivity, reliability, and specificity [11]. Therefore, it is urgent to develop new methods for the rapid, sensitive, portable, and highly specific detection of protein biomarkers.

The CRISPR/Cas system is an adaptive immune system that originated from prokaryotes consisting of CRISPR sequences (Clustered Regularly Interspaced Short Palindromic Repeats) and proximity CRISPR-associated protein (Cas proteins), which can effectively and accurately identify and cleave foreign nucleic acids. This results in silencing their expression, and maintaining the stability of its genetic system through three stages of adaptation, recognition, and interference, thus effectively defending against foreign genes (e.g., phages and exogenous plasmids) [12,13]. In 1987, the CRISPR system was first discovered [14], which was subsequently coined as the CRISPR system in 2002 [15]. The CRISPR/Cas system has the advantages of programmability, specificity, sensitivity, and single-base resolution for nucleic acid recognition, and is now widely used in the biomedical fields [16,17,18]. It is generally divided into two types according to the structure of Cas proteins: Class I Cas proteins are effector complexes composed of multiple subunits; and class II Cas proteins are single effector proteins including Cas9, Cas12a, Cas13a, and Cas14 systems [19]. In recent years, some class II Cas proteins have been found to exhibit excellent signal amplification to neighboring non-target ssDNA or RNA with high non-specific cleavage efficiency [20,21]. For example, the Cas12a protein can specifically recognize the Protospacer Adjacent Motif (PAM) sequence of target DNA under the guidance of CRISPR RNA (crRNA) through forming a Cas12a/crRNA/DNA ternary complex, exhibiting non-specific cleavage activity to nearby ssDNA (trans cleavage activity) [22,23]. Unlike Cas12a, Cas13a is an RNA-mediated RNA endonuclease containing two HEPN structural domains that specifically recognize and cleave target RNA under the guidance of crRNA [24,25], which is able to indiscriminately cleave nearby RNA [26].

Due to the characteristics above-mentioned, CRISPR/Cas systems have been explored in the field of nucleic acid detection [27,28]. In 2017, Zhang’s team reported the seminal work of a CRISPR/Cas13a-based ultra-sensitive and specific method for the rapid detection of DNA and RNA, called Specific High-Sensitivity Enzymatic Reporter Unlocking (SHERLOCK), by combining the trans cleavage activity of the Cas13a protein with a fluorescently dual-labeled signal reporter [29]. Later on, Doudna’s team also conducted a landmark work of dubbed DNA Endonuclease Targeted CRISPR Trans Reporter (DETECTR) based on the CRISPR/Cas12a system, enabling nucleic acid detection at the attomolar level and the detection of two types of human papillomavirus (HPV), 16 and 18, in clinical samples [30]. Now, CRISPR technology has achieved remarkable success in the field of molecular diagnosis [31], which has recently become a hot topic in COVID-19 diagnosis [32,33]. Moreover, CRISPR diagnostic has been expanded for the detection of other substances such as ions [34,35], proteins [36], small molecules [37,38], etc., largely compensating for the limitations of traditional molecular diagnosis technology. For proteins, as the CRISPR/Cas system is only capable of recognizing and cleaving nucleic acid sequences, protein targets cannot directly activate the trans cleavage activity of Cas endonuclease, so it needs to convert the information of the protein molecule into the detectable nucleic acid signal that can be responded by CRISPR/Cas systems.

**Scheme 1:**
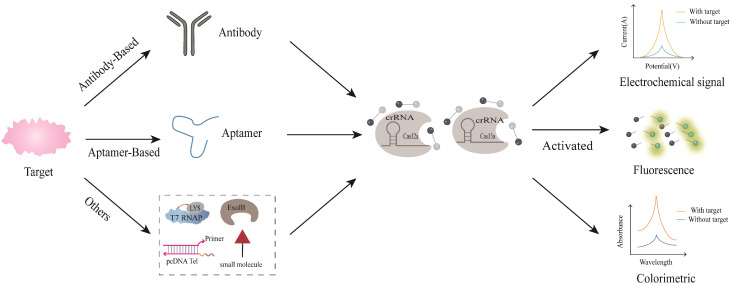
A schematic illustration of CRISPR/Cas-based biosensors for protein detection.

To date, initial efforts have been made for the detection of protein biomarkers based on CRISPR/Cas12a systems due to the desirable characteristics of CRISPR diagnostics. However, most of the currently published reviews are about the research progress of CRISPR/Cas system applications in gene editing [39,40], gene therapy [41], bioimaging [42,43], pathogen diagnosis [44,45], and nucleic acid detection [46,47,48]. Although there are few reviews describing CRISPR-based biosensors for non-nucleic-acids, the protein detection section is not the main part, lacking a comprehensive and systematic summary on CRISPR-based protein detection [48,49,50]. A comprehensive literature review on the application of CRISPR/Cas systems in protein detection can provide a better understanding of CRISPR/Cas system applications in biosensing. Therefore, in this review, we present recent advances in CRISPR-based biosensors for protein analysis, which can be divided into three aspects based on signal conversion: antibody-assisted CRISPR/Cas-based protein detection, aptamer-assisted CRISPR/Cas-based protein detection, and miscellaneous CRISPR/Cas-based methods for protein detection, respectively (Scheme 1). We introduce their applications in protein detection with recent examples, and discuss their advantages, significance, and drawbacks. Finally, their challenges and potential for future applications are also discussed.

## 2. Antibody-Assisted CRISPR/Cas-Based Protein Detection

The antibody is a major recognition biomolecule with a symmetrical structure of two heavy chains (H chain) and two light chains (L chain) connected by disulfide and non-covalent bonds, capable of specifically recognizing and binding to antigenic determinants on the surface of the target proteins with high affinity and specificity [51,52]. The structure of the entire antibody molecule can be divided into two parts: the variable region (V region) and the constant region (C region) [53]. Antibodies are routinely used as biological recognition elements for proteins in immunosensors, which are currently the most important and widely used biosensors for protein detection [54]. The traditional ELISA method is well-recognized as the gold standard for protein detection [55], and its detection principle is based on the formation of a sandwich antibody–antigen–antibody structure, in which the enzyme (usually horseradish peroxidase (HRP)) labeled on the antibody induces the enzymatic signal amplification for measuring the concentration of targets. However, it is still not sensitive enough for the rapid detection of ultralow concentrations of protein biomarkers [56,57]. Moreover, the labeling of enzymes to antibodies usually requires complicated chemical modification and purification, easily resulting in the degradation of enzymes or antibodies. On the other hand, due to the high programmability, trans cleavage activity, and specificity of CRISPR systems with excellent signal amplification [58,59], the CRISPR/Cas systems have been combined with immunoassays for protein detection with significantly improved sensitivity. It was found that most of them were based on antibody–antigen–antibody type sandwich assays, and one was based on antigen–antibody recognition with the proximity CRISPR/Cas12a assay (Table 1).

**Figure 1:**
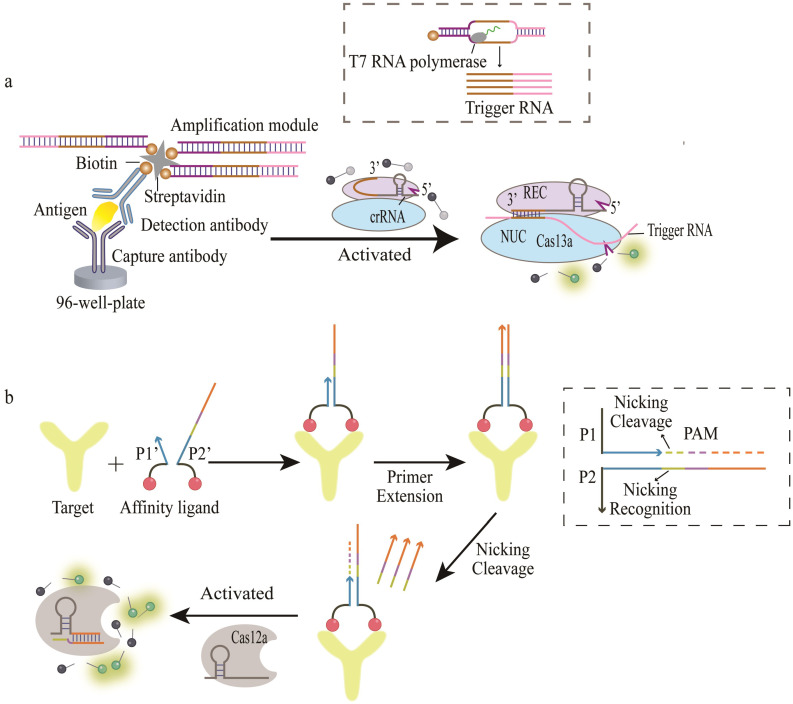
The strategies for antibody-based CRISPR biosensors for protein detection. (**a**) Schematic illustration of the CRISPR/Cas13a signal amplification linked immunosorbent assay (CLISA). Reproduced with permission from [61]. Copyright 2020 American Chemical Society. (**b**) Schematic illustration of the detection of antibodies using proximity CRISPR/Cas12a integrated with nicking cleavage. Reproduced with permission from [63]. Copyright 2021 Royal Society of Chemistry.

Li et al. reported a universal CRISPR-based immunosignaling enhancer called CRUISE, which constructed an antibody-ssDNA (Abs-ssDNA) through streptavidin-biotin binding to a biotinylated single-stranded DNA (ssDNA) [60]. Abs-ssDNA can act as a primary antibody to directly capture the target protein, and also indirectly as a secondary antibody to recognize the Fc fragment of the antibody used, forming a typical sandwich structure. Both methods can be used after washing away the unbound Abs-ssDNA, and the bound Abs-ssDNA activates the trans cleavage activity of CRISPR/Cas12a, cleaving the reporter molecule for the generation of a fluorescence signal. Secondary antibodies were integrated into a variety of different immunoassays to relieve the need of redundant recognition elements other than antibodies, providing three orders of magnitude higher sensitivity than traditional ELISA methods for IFN-γ detection. However, the CRUISE system has limitations similar to those of traditional ELISA methods such as the non-specific binding of Abs-ssDNA couplers that can reduce the analytical performance of the system and require better blocking strategies. Careful optimization of the antibody capture fixation method for 96-well plates is required to achieve higher sensitivity and specificity. Based on the classical sandwich-type sandwich structure, Chen et al. proposed a CRISPR/Cas13a signal amplification correlation immunosorbent assay called CLISA by designing biotinylated dsDNA containing a T7 promoter sequence instead of the traditional enzyme used for signal output (generally horseradish peroxidase) (Figure 1a) [61]. The presence of the target captures the secondary antibody on the capture antibody attached on the 96-well plate. The secondary antibody subsequently ligates the biotinylated dsDNA through biotin–streptavidin interaction, while the captured dsDNA is transcripted to generate a large amount of trigger RNA under T7 RNA polymerase. The trigger RNA activates CRISPR/Cas13a system under the assistance of crRNA along with the generation of fluorescence for protein detection. The CLISA detected human interleukin 6 (IL-6) with a limit of detection (LOD) of 45.81 fg/mL (2.29 fM) and human vascular endothelial growth factor (VEGF) with a LOD of 32.27 fg/mL (0.81 fM). It should be noted that a strict RNase-free environment is required for CLISA due to the use of RNA as the signal output. To improve the detection sensitivity, Lee and coworkers introduced antibody–DNA barcode conjugates with multiple Cas12a recognition sites into the conventional sandwich assay system using the affinity of biotin–streptavidin [62]. The detection signal was doubly amplified by increasing the number of Cas12a recognition sites on the DNA barcodes and the trans-cleavage activity of the CRISPR system. The assay achieved the detection of chemokine ligand 9 (CXCL9) in urine without PCR amplification, displaying a LOD of 14 pg/mL, which was seven times higher than that of the conventional ELISA method. The authors successfully evaluated CXCL9 protein in the urine of 11 kidney transplant patients with a 100% detection rate using this method, providing a potential tool for the non-invasive clinical diagnosis of kidney transplant rejection.

To improve the sensitivity and binding specificity, Li et al. developed a universal proximity CRISPR/Cas12a assay by cleverly designing two target-specific primers with different lengths, P1 and P2, which were modified with affinity ligands that bind to different antigenic epitopes of the same antibody (Figure 1b) [63]. When the target is present, P1 and P2 are in proximity to each other. The primer extension reaction is triggered to generate a stable dsDNA containing the PAM sites, while the P1 is cleaved off using a nicking nuclease during the extension, which serves as the crRNA of the CRISPR/Cas12a system, to activate the trans cleavage activity of Cas12a, thus generating fluorescence and enabling the detection of the antibody at low concentrations of 1 pM. After further optimization, Li and coworkers reported that an improved iPCCA assay system achieved a LOD of IL-6 as low as 100 fM. This method can be applied in homogeneous solutions while maintaining detection sensitivity and does not require complex fixation and washing steps.

## 3. Aptamer-Assisted CRISPR/Cas-Based Protein Detection

Due to the large molecular mass, high immunogenicity, and batch-to-batch variations of antibodies, the reliability and repeatability of antibody-based CRISPR biosensors may vary for each test, limiting their applications in protein detection. In the last decades, nucleic acid aptamers (simplified aptamers) have received the widespread attention of scientists, thanks to their excellent performance in sensing platforms, low cost, and comparable sensitivity [64]. Aptamers are synthetic functionalized single-stranded oligonucleotide sequences (DNA and RNA), also known as chemical antibodies, which are specific nucleic acid sequences and have three-dimensional structures, allowing them to bind target molecules with high affinity and specificity [65,66]. In contrast to antibodies, aptamers show the advantages of low immunogenicity, low preparation cost, long-term storage, ease of modifications, high stability, insensitivity to temperature, small size, no inter-batch variation, easy combination with nucleic acid signal amplifications, and applicability to a wide range of targets [67,68]. Aptamers are generally selected from nucleic acid molecular libraries by the Systematic Evolution of Ligands by EXponential enrichment (SELEX) [69,70], which was originally proposed by Tuerk and Ellington in 1990 [71]. The SELEX technology has successfully identified various aptamers for a range of proteins, which are widely used in cancer diagnosis, bioimaging, and therapy [72,73].

### 3.1. With Nucleic Acid Amplification

As the level of proteins is low in clinical samples, it usually needs to amplify the target proteins to effectively detect proteins. Most of the reported methods for protein amplifications depend on isothermal amplification such as hybrid chain reaction (HCR) [74], strand displacement amplification (SDA), [75,76], etc. In addition, traditional polymerase chain reaction (PCR) is also applied [77,78] due to its high sensitivity. Further powered by the amplification function of the CRISPR/Cas system, the integration of nucleic acid amplifications to CRISPR/Cas systems can achieve much improved sensitivity (Table 2).

**Figure 2:**
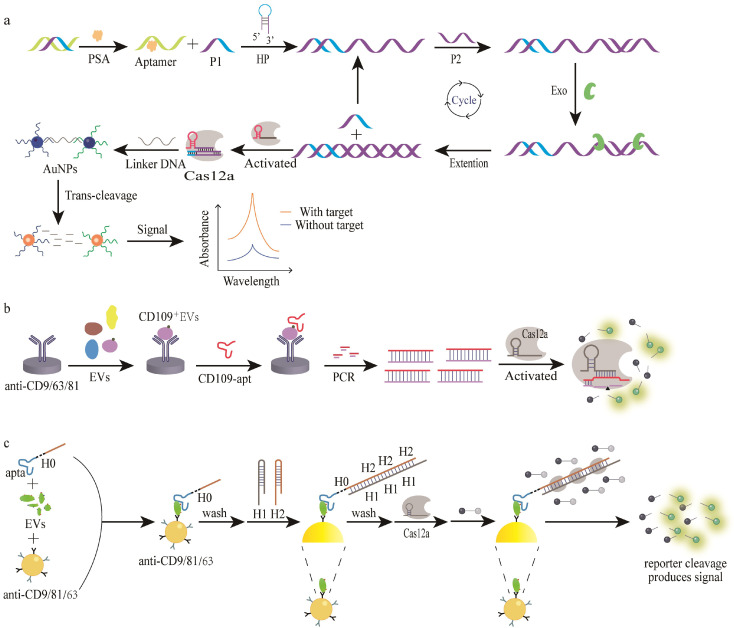
The strategies for the aptamer-assisted CRISPR/Cas-based detection of protein with nucleic acid amplification. (**a**) A schematic diagram of the nicking enzyme-free SDA-assisted CRISPR/Cas colorimetric detection of PSA. Reproduced with permission from [83]. Copyright 2022 Elsevier. (**b**) A schematic illustration of the aptamer-CRISPR/Cas12a assay with PCR amplification. Reproduced with permission from [87]. Copyright 2021 Elsevier. (**c**) A schematic diagram of the detection of TEV surface proteins by apta-HCR-CRISPR. Reproduced with permission from [89]. Copyright 2022 Elsevier.

Prostate-specific antigen (PSA) is a serine protease produced by prostate epithelial cells, and its level is generally very low in normal human serum, but is abnormally high in the serum of prostate cancer patients [79,80]. Therefore, PSA is the most important prostate cancer biomarker, where its diagnostic specificity can reach more than 90% [81]. The Wang group developed a nicking enzyme-free SDA-assisted CRISPR/Cas12a-based colorimetric method for the detection of PSA with a LOD of 0.030 ng/mL (Figure 2a) [82]. When the PSA target is present, the released ssDNA opens the hairpin structure of the HP to release complementary ssDNA, triggering a nicking enzyme-free SDA reaction. The generated dsDNA serves as an activator of the CRISPR/Cas12a system to activate the trans cleavage activity of the Cas12a endonuclease, which non-specifically cleaves the nearby AuNP-linker probe. This allows the AuNPs to change from an aggregated purple state to a dispersed red state, which is colorimetrically determined, along with a visual readout. In this work, the exonuclease polymerase is harnessed for releasing cDNA from HP-cDNA during SDA, along with triggering the next SDA cycle, this unique design renders the biosensor simpler and more convenient for clinical testing. The same group also reported a colorimetric assay for serum PSA using the nonenzymatic and isothermal properties of HCR to convert serum PSA into nucleic acid products [83]. The presence of PSA triggers HCR amplification to produce dsDNA containing multiple PAM sites recognized by Cas12a, activating Cas12a’s trans cleavage activity, which nonspecifically cleaves the DNA–AuNP probe pairs along with a colorimetric signal. This strategy enables the sensitive and selective detection of PSA with a LOD of 0.10 ng/mL in both the spiked and clinical samples.

Ultrasensitive detection of tumor-derived extracellular vesicles (TEVs) is key for the prognosis and diagnosis of cancers [84,85]. Li et al. developed a PCR-powered-CRISPR/Cas12a assay, which consists of three parts: aptamer recognition, PCR amplification, and CRISPR/Cas12a detection (Figure 2b) [86]. The aptamers for membrane proteins were coated on microtiter plates, which can specifically recognize and bind to extracellular vesicle surface membrane proteins for the formation of a sandwich-type complex. After washing away the unbound aptamers, the bound aptamers were amplified by PCR to generate a large amount of dsDNA, which activates the trans cleavage activity of Cas12a, enabling the detection of CD109^+^ and EGFR^+^ TEV at a concentration as low as 100 particles/mL. Moreover, the linear range spans six orders of magnitude (10^2^–10^8^ particles/mL), which is sufficient to detect TEVs in low volume (50 μL) samples. However, this method uses PCR amplification strategy, and its thermal cycling process requires complex instrument control, which limits its clinical application. Moreover, high temperature during the thermal cycling process may denature the proteins, affecting the sensing performance. Zhao et al. also reported an HCR amplified CRISPR/Cas12a-based biosensor, named AID-Cas, for the wash-free detection of EVs in the concentration range of 10^2^–10^6^ particles/μL [87]. The CD63 aptamer structural domain contained on the variant probe specifically recognizes and binds CD63^+^ TEVs, triggering double-loop HCR amplification. The amplified dsDNA contains a T7 promoter recognition sequence, which can be recognized by T7 RNA polymerase and transcribed to a large amount of RNA, serving as the crRNA of the CRISPR/Cas12a system. This method enables the quantitative detection of TEVs in the cell culture supernatants and clinical samples. However, the free CD63 protein from ruptured EVs or cells may interfere with the assay results. Similarly, Xing et al. developed an apta-HCR-CRISPR assay for the ultra-sensitive quantification of TEV surface proteins, which uses HCR to amplify the TEV surface proteins based on the corresponding aptamers, generating dsDNA containing multiple PAM sites for activating the trans cleavage activity of Cas12a in the presence of crRNA [88] (Figure 2c). This method can directly be used for the clinical analysis of circulating TEVs in 50 μL serum, achieving TEV detection at a concentration as low as 10^2^ particles/µL in complicated biological samples.

**Table 2:** A comparison of aptamer-assisted CRISPR/Cas-based biosensors for protein detection with nucleic acid amplification.

Method	Target	LOD	Detection Range	Signal	Refs.
Nicking enzyme-free SDA-assisted CRISPR/Cas12a	PSA	0.030 ng/mL	0.1–5 ng/mL	Colorimetric	[82]
Nonenzymatic HCR-powered CRISPR/Cas12a	PSA	0.10 ng/mL	0.2–4.0 ng/mL	Colorimetric	[83]
PCR-powered CRISPR/Cas12a	CD109^+^ and EGFR^+^ TEVs	100 particles/mL	102–108 particles/mL	Fluorescence	[86]
AID-Cas	CD63-positive EVs	102 particles/µL	102–106 particles/μL	Fluorescence	[87]
apta-HCR-CRISPR	TEV	102 particles/µL	64–106 particles/µL	Fluorescence	[88]

### 3.2. Amplification-Free

The amplification of target proteins often requires complicated pre-detection processing, which often leads to problems such as non-specific amplification or reagent contamination [89,90]. To overcome these problems, a series of amplification-free sensing strategies have been proposed for protein analysis such as multiple activator dsDNA, the use of ssDNA blockers, the combination with electrochemical signal amplification, multiple Cas recognition sites, and other method-coupled CRISPR/Cas techniques (Table 3).

**Figure 3:**
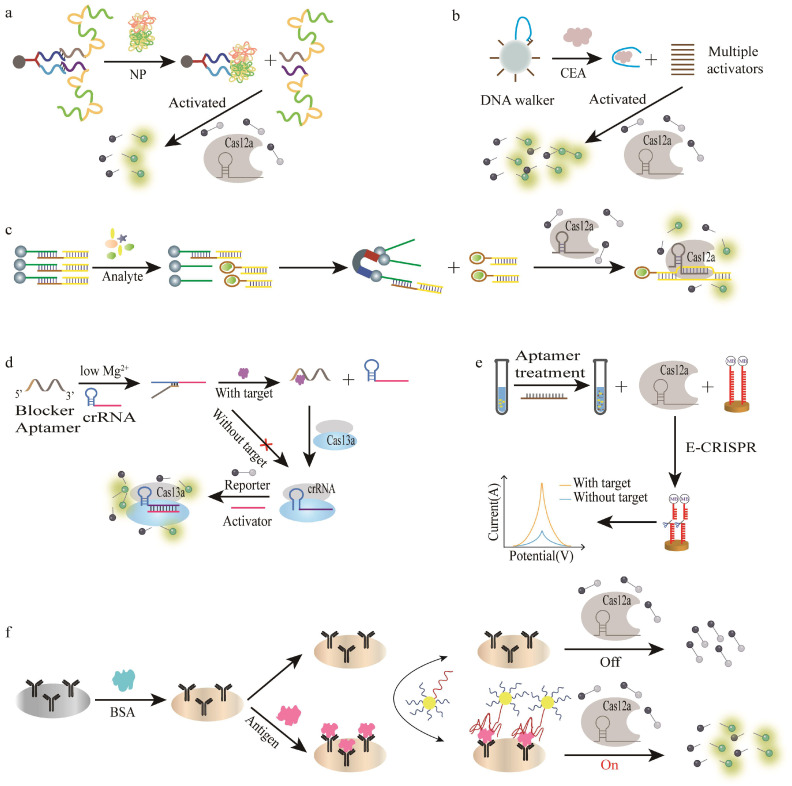
The strategies for aptamer-assisted CRISPR/Cas biosensors for protein detection without amplification. (**a**) A schematic diagram of the dual aptamer-based CRISPR/Cas12a biosensor for synergistic sensing of SARS-CoV-2 antigen detection without PCR amplification. Reproduced with permission from [92]. Copyright 2021 Elsevier. (**b**) A schematic diagram of the DNA walker amplified “one-to-many” CRISPR/Cas12a-mediated fluorescent biosensor for detecting CEA. Reproduced with permission from [93]. Copyright 2022 Elsevier. (**c**) A schematic diagram of a multifunctional biosensing platform combining CRISPR/Cas12a and the aptamer for detecting AFP. Reproduced with permission from [94]. Copyright 2021 Elsevier. (**d**) A schematic illustration of the regulation of the trans-cleavage activity of CRISPR/Cas13a by the ssDNA blocker at low Mg^2+^ concentration for protein detection. Reproduced with permission from [95]. Copyright 2022 American Chemical Society. (**e**) A schematic illustration of E-CRISPR for protein detection. Reproduced with permission from [96]. Copyright 2019 John Wiley and Sons. (**f**) A schematic illustration of Nano-CLISA for protein detection. Reproduced with permission from [112]. Copyright 2021 Elsevier.

Zhao et al. designed a dual aptamer sensor to implement a multi-trigger dsDNA tandem binding CRISPR/Cas12a system for the PCR-free detection of the SARS-CoV-2 antigenic nucleocapsid protein (Np) (Figure 3a), in which a hybrid DNA containing Cas12a-triggered dsDNA (HyDNA) modified with two aptamers, A48 and A61, was used to recognize different epitopes of Np [91]. When Np is present, the aptamers release two HyDNA activators from the hybrid DNA, subsequently activating Cas12a’s trans cleavage activity for non-specifically cleaving a nearby fluorescently labeled ssDNA probe along with the generation of the fluorescence signal. This multi-trigger dsDNA tandem element may be able to serve as a versatile tool for implementing highly sensitive CRISPR biosensors. Similarly, Li et al. used a DNA walker to amplify “one-to-many” by embedding the target’s aptamer in the locked strand and then hybridizing it with the walking strand [92] (Figure 3b). The walker is efficiently driven by using a nicking endonuclease as the energy supply. When the target is present, the walker can compete for the release of the walking strand to generate multiple activators, thus activating the trans cleavage activity of Cas12a to generate a fluorescent signal. To validate the performance of the method in real samples, the authors applied it to the detection of inactivated SARS-CoV-2 in the saliva and serum spiked samples, with a positive detection rate of 100%. The LOD for carcinoembryonic antigen is 0.32 pg/mL and features a fluorescent reporter gene loaded onto a biochip coated with photonic crystals (PC) and excited by a mini-type portable blue light, allowing the results to be observed with only a smartphone without the need for other sophisticated imaging tools. Zhao et al. immobilized biotinylated ssDNA partially bound to the designed aptamer-dsDNA complex on streptavidin-coated magnetic beads (MBs), in which the target can bind to the aptamer to release the dsDNA [93] (Figure 3c). The released dsDNA is used as an activator of the CRISPR/Cas12a system, triggering the trans cleavage activity of Cas12a, and thus non-specifically cleaving the fluorescently labeled ssDNA signal probe with a fluorescent signal output. This enabled the analysis of the tumor biomarker alpha-fetoprotein (AFP) in less than 20 min with a LOD as low as 0.07 fM/L, while the quantitative analysis of cocaine was at a LOD of 0.34 μmol/L. This highly modular biosensing platform has great potential for the detection of other analytes.

Under the condition of very low magnesium ion (Mg^2+^) concentration, Liu et al. broadened the biosensing application of CRISPR/Cas13a by introducing an ssDNA blocker modified with an aptamer at the end to program the trans cleavage activity of CRISPR/Cas13a [94] (Figure 3d). The ssDNA blocker binds to crRNA and blocks the conformational change of crRNA to inhibit the trans cleavage activity of Cas13a. The presence induces the ssDNA blocker to release crRNA, restoring the Cas13a’s trans cleavage activity along with enhanced fluorescence signal. Moreover, this strategy is applicable to the detection of analytes that can bind to the ssDNA blocker to release crRNA. In addition, it was found that the Mg^2+^ concentration plays an important role in the activity of the Cas13a protein. If the Cas13a protein is highly active, its enzyme activity will not be easy to block, resulting in high fluorescence background, so a suitable Mg^2+^ concentration is demanded to block its activity effectively. Controlling the structure of crRNA by a simple ssDNA blocker to regulate the trans cleavage activity of Cas13a offers new opportunities for the development of CRISPR/Cas13a biosensors.

In 2019, Liu’s team combined electrochemistry with the CRISPR system to develop an aptamer-based E-CRISPR tandem technology for protein detection for the first time [95] (Figure 3e). The authors validated the detection performance of the E-CRISPR electrochemical biosensor using the growth factor beta 1 (TGF-*β*1) protein by square wave voltammetry (SWV). The ssDNA aptamer also serves as the template for activating the trans cleavage activity of Cas12a, so the aptamers activate the CRISPR/Cas12a system without the addition of TGF-*β*1, cleaving the ssDNA reporter (methylene blue-labeled) with no electrochemical signal output. In contrast, the presence of TGF-*β*1 weakens the trans cleavage activity with more intact ssDNA reporters and a stronger electrochemical signal. The linear range was up to three orders of magnitude, with a LOD of 0.2 nM, while the detection was completed in 60 min. Very recently, Yuan et al. reported the CRISPR/Cas12a coupled voltage enrichment by coupling electrochemical and CRISPR systems [96]. The authors designed two aptamers, Apt_VEGF-HBD_-T18-MB and Apt_VEGF-RBD_, which can specifically recognize the HBD and RBD domains of vascular endothelial growth factor (VEGF), respectively. Among them, Apt_VEGF-HBD_-Tx-MB consists of a thiol group at the 5′ end and a different internal T (Tx) site or MB tag at the 3′ end of the aptamer, which is covalently modified to AuNPs@Ti_3_C_2_T_X_Mxene/GCE through Au–S bonding. When the target is present, Apt_VEGF-HBD_-Tx-MB is recognized and bound to it, bringing the MB close to the electrode surface, improving the electron transfer efficiency, and generating a “signal-on” response; when the target is not present, the MB is away from the electrode surface, resulting in the “signal-off” response; when a positive voltage of 0.4 V was applied, the negatively charged MB groups were rapidly attracted to the electrode surface, resulting in a stronger current signal, resulting in a “signal superconducting” response. This strategy goes through a “signal on–off–on” sandwich-type mode for the detection of VEGF rather than a complicated target amplification step to enrich the cleaved signal probe. Converting the “signal-off” of CRISPR/Cas12a cleavage to “signal super on” further improves the current response, thereby simplifying the routine detection process and amplifying the electrochemical signal. The linear range of VEGF detection was from 1 pM to 10 μM in the serum samples, with a LOD of 0.33 pM.

Electrochemiluminescence (ECL) is chemiluminescence that originates from the electron transfer between species generated on the surface of electrodes [97,98], which simultaneously has the dual advantages of electrochemical analysis and chemiluminescence such as high sensitivity, good reliability, simple operation, and fast analysis process [99,100], so it has been widely used in the biomedical field [101,102], food safety assessment [103,104], environment monitoring [105,106], and other fields of biomolecule detection [107,108]. Based on the merits of ECL, Liu et al. combined the advantages of spherical nucleic acids with CRISPR technology, in which a Y-shaped DNA structure constructed from helper DNA (A1), prostate cancer biomarker α-methylacyl coenzyme A racemase (AMACR) adaptors, and DNA activators are loaded onto gold nanoparticle-modified Fe_3_O_4_ magnetic beads (Au@Fe_3_O_4_MBs) [36]. Y-SNA serves as a target transducer to convert the protein signal into the programmable nucleic acid signal, while 1-pyrenecarboxaldehyde (Pyc) as a nanoemitter is embedded in magnetic mesoporous silica nanoparticles (MMSNs). Meanwhile, silver nanoparticles (AgNPs) serve as a co-reaction gas pedal to synergize with Pyc, and the synthesized AgNP-Pyc@MMSNs nanomaterial has a strong and stable ECL signal. The presence of the target protein induces the release of the DNA activator of Cas12a, activating the trans cleavage activity of Cas12a and thus non-specifically cleaving the ferrocene-labeled quenching probe (QP) in its vicinity with the ECL signal output. The designed ECL biosensor was used to determine AMACR from 10 ng/mL to 100 μg/mL, with a LOD of 15.8 pg/mL. However, the complicated pre-processing step causes difficulties in the large-scale production of biosensor-related reagents for further clinical applications, and the vulnerability to protease denaturation during the preparation process dampens the performance of the biosensors.

**Table 3:** A comparison of the aptamer-based sensors without targeted amplification for protein detection by CRISPR.

Method	Target	LOD	Detection Range	Signal	Refs.
Dual aptamer-assisted CRISPR/Cas12a	SARS-CoV-2 antigen	0.17 fM, two copies/μL	0.19–781 pM	Fluorescence	[91]
DNA walker-amplified CRISPR/Cas12a	CEA	0.32 pg/mL	0.7 pg/mL–1 ng/mL	Fluorescence	[92]
Functional MBs-assisted CRISPR/Cas12a	AFP	0.07 fM	0.24–977 fM	Fluorescence	[93]
ssDNA blocker-assisted CRISPR/Cas13a	Enzymes, antigens/proteins, and exosomes	-	-	Fluorescence	[94]
E-CRISPR	TGF-β1	0.2 nM	-	Electrochemical signal	[95]
Voltage enrichment-coupled CRISPR/Cas12a	VEGF	0.33 pM	1 pM–10 μM	Electrochemical signal	[96]
Spherical nucleic acids-assisted CRISPR/Cas12a	AMACR	1.25 ng/mL	10 ng/mL–100 μg/mL	ECL	[36]
ALCIA	PDGF-BB	550 aM	-	Fluorescence	[109]
CAFI	Cytokine IFN-γ	58.8 aM	1 fg/mL–100 pg/mL	Fluorescence	[110]
Nano-CLISA	CEA and PSA	13.9 fg/mL and 5.6 fg/mL, respectively	0.6–120 ng/mL and 0.5–150 ng/mL, respectively	Fluorescence	[111]

Li et al. proposed a novel aptamer-based CRISPR/Cas12a immunoassay method called ALCIA, which established a link between non-nucleic acid targets and the CRISPR/Cas12a system by modifying the analyte-targeted aptamer (named Apt-acDNA) at the 5′ end of the activator DNA (acDNA), which can activate the trans cleavage activity of Cas12a upon target recognition, along with the fluorescence signal [109]. The authors designed dual aptamers based on two identical subunits of platelet-derived growth factor BB (PDGF-BB), where one aptamer was modified on the plate substrate to capture PDGF-BB and the other was modified at the 5′ end of acDNA to release the activator DNA. When PDGF-BB is present, it can form a sandwich-like structure of aptamer/PDGF-BB/Apt-acDNA for activating Cas12a. This assay detects PDGF-BB in the serum, urine, and saliva during a narrow range of 0–150 pM, with a LOD of 1.57 pM. The output signal of ALCIA can be adapted based on the actual needs. Moreover, its sensing principle is similar to ELISA and is highly compatible with traditional ELISA methods, which has the great potential for bioanalytical analysis and clinical testing. Similarly, Deng et al. developed a CRISPR/Cas12a-assisted fiber-optic immunosensor (CAFI) that could detect IFN-γ in the serum, urine, and saliva with a LOD of 1 fg/mL (58.8 aM) over a detection range of 1 fg/mL to 100 pg/mL [110]. By modifying biotinylated capture antibodies on the surface of antifouling glass fibers modified with silane-polyethylene glycol-biotin and streptavidin, an antibody–analyte–adaptor sandwich structure can be formed in the presence of the target. The CAFI assay system can be applied for other analytes such as insulin detection and analysis by simply changing the aptamer and capturing antibodies. However, its detection time takes 4 h, which hampers its on-site applications. Increasing the temperature of the reaction to 37 °C may be a feasible solution in reducing the detection time while maintaining sensitivity. Zhao et al. covalently modified aptamer and Cas12a target DNA activators on AuNPs to form sandwich-like structures of antibody–target–aptamers [111]. When the target is present, the sandwich-like structure forms, while the activators modified on AuNPs are captured for activating the trans-cleavage activity of Cas12a, thus cleaving ssDNA modified with a fluorophore (FAM) and a quencher (BHQ1) at both ends, along with an increasing fluorescence signal (Figure 3f). The reported Cas12a/crRNA-based nano-immunosorbent assay (Nano-CLISA) can determine carcinoembryonic antigen (CEA) and PSA in clinical samples with the LODs of 13.9 fg/mL and 5.6 fg/mL, respectively. AuNP-modified oligonucleotides to activate CRISPR/Cas12a can greatly enhance the sensitivity of the assay, which makes the assay 1000 times sensitive than conventional ELISA methods.

## 4. Miscellaneous CRISPR/Cas-Based Methods for Protein Detection

In addition to protein signal conversion methods based on well-recognized antibodies and aptamers, some other signal conversion methods have also been reported such as the use of PAM-free conditional DNA substrate (pcDNA), protection experiments with the help of nucleic acid exonuclease III, the use of protease-activated RNA polymerase, and the use of small molecule modification activator ssDNA (Table 4).

**Figure 4:**
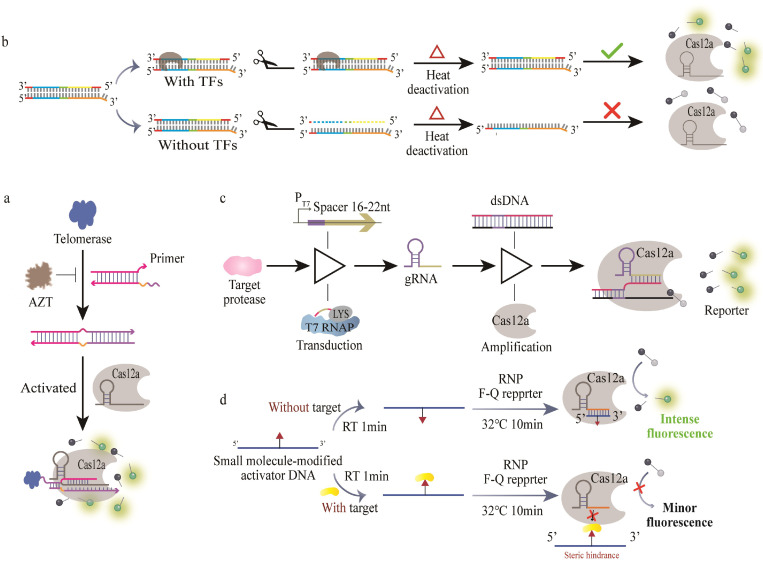
The strategies for the miscellaneous CRISPR/Cas-based methods for protein detection. (**a**) A schematic illustration of pcDNA-based Cas12a for detecting telomerase activity in living cells. Reproduced with permission from [117]. Copyright 2021 Royal Society of Chemistry. (**b**) A schematic diagram of ExoIII-protected CRISPR/Cas12a-based biosensor for detecting TFs in cancer cells. Reproduced with permission from [120]. Copyright 2021 Elsevier. (**c**) A schematic diagram of the PR-Cas detection of proteases. Reproduced with permission from [121]. Copyright 2020 Springer Nature. (**d**) A schematic diagram of a strategy to recognize protein/small molecule interactions based on CRISPR/Cas12a trans cleavage activity. Reproduced with permission from [122]. Copyright 2021 Elsevier.

The CRISPR/Cas system has become a powerful tool for live cell imaging, but its utility is limited to genomic loci and mRNA imaging in living cells [112,113]. On the other hand, the recognition of the CRISPR/Cas12a system dsDNA heavily depends on PAM sequences [114], which greatly limits the detection scope of targets. The design of DNA substrates using a universal response mechanism can expand the types of analytes. Inspired by the fact that the Cas12a/gRNA complex can recognize unwound DNA substrates without the restriction of PAM [115], the Nie team rationally constructed the unwound seed region and introduced a bubble structure in the seed region to make it unwind to overcome the CRISPR/Cas12a system’s PAM limitation (Figure 4a) [116]. By designing a pcDNA, the target recognizes and converts the corresponding pcDNA into a PAM-free dsDNA substrate (pDNA). pDNA activates the nuclease activity of Cas12a to nonspecifically cleave the surrounding fluorescent ssDNA signal probe for living cell imaging, and this PAM-free strategy was found to be suitable for adenosine triphosphate (ATP), miRNA, and telomerase. This biosensor can also be applied for the sensitive sensing of a wide range of biomolecules such as intracellular enzymes, small molecules, and microRNAs. The main limitation of this biosensor is that its reaction kinetic depends on the effective collision of reactants in the cytoplasm, which leads to reduced sensitivity and reproducibility due to the complicated biological environment inside the cells. Encapsulating the components of the sensing system into a restricted space by DNA technology or liquid–liquid phase separation may be an effective solution to alleviate this limitation.

Cheng et al. also reported a AuNP-assisted CRISPR/Cas system for the visual detection of telomerase activity in three cases: positive (P), negative (N), and false-negative (FN) [117]. The authors designed telomeric repetitive sequence DNA and internal control crRNAs, crRNA1 and crRNA2, respectively. Both Cas12a/crRNA1 and Cas12a/crRNA2-mediated assays in the positive state keep AuNPs in the dispersed state. Cas12a/crRNA1-mediated assays in the negative state induce the cross-linking of AuNPs, and Cas12a/crRNA2-mediated assays ensure that the AuNPs remain dispersed. The false-negative state due to the PCR inhibitor or telomere repeat amplification protocol (TRAP) reagent errors allowed for both the Cas12a/crRNA1 and Cas12a/crRNA2-mediated analysis to induce the cross-linking of AuNPs. The platform was able to visually identify false-negative results caused by PCR inhibitor and TRAP reagent errors free of a complicated polyacrylamide gel electrophoresis (PAGE) process, significantly improving the accuracy of conventional TRAP. The authors also validated that the Cas9-mediated TL-LFA platform can also be used for accurate telomerase activity detection, which can be achieved within 15 min on a single test strip.

Exonuclease III (ExoIII) recognizes flat-ended dsDNA and cleaves it from 3′ to 5′ to produce ssDNA with a 3′ protruding end [118]. An ExoIII-assisted Cas12a biosensing system is reported for the detection of transcription factors (TFs) based on which the activator dsDNA of Cas12a also contains the structural domain of TFs [119] (Figure 4b), in which TFs can bind to the activator to prohibit the degradation of the dsDNA by ExoIII. The intact dsDNA activator is thermally inactivated at 65 °C, which is further used to activate the trans cleavage activity of Cas12a. This method is applied for the detection of the nuclear factor-κB (NF-kB) p50 subunit with a LOD of 0.2 pM. The method has the potential to screen TF inhibitors and evaluate their biological activities. However, it should be noted that the method is limited to its long detection time and requires temperature control. Moreover, the assay performance is affected by the cellular nuclear protein extracts.

**Table 4:** A comparison of the miscellaneous CRISPR/Cas-based methods for protein detection.

Method	Target	LOD	Detection Range	Signal	Refs.
PAM-free CRISPR/Cas12a	Telomerase	-	-	Fluorescence imaging	[116]
AuNPs-assisted CRISPR/Cas12a	Telomerase	-	-	Colorimetric	[117]
ExoIII-assisted Cas12a	TFs	0.2 pM	0.5–1600 pM	Fluorescence	[119]
PRs-assisted CRISPR/Cas12a	MMP-2 and thrombin	5.4 fM and 47.8 fM, respectively	10 fM–0.5 nM and 100 fM–0.5 nM, respectively	Fluorescence	[120]
AD-assisted CRISPR/Cas12a	Streptavidin/biotin and antidigoxin/digoxin interaction	0.03 nM and 0.09 nM, respectively	0.1–2.5 nM and 0.2–5 nM, respectively	Fluorescence	[121]

By using protease-activatable RNA polymerases (denoted as PRs), Yang et al. transformed protein hydrolysis events into multiple programmable RNA sequences by in vitro transcription using PRs as transducers, and protease hydrolysis can activate RNA polymerase transcription to produce RNA, which serves as a guide RNA (gRNA) for activating the CRISPR/Cas12a system in the presence of template dsDNA, resulting in a corresponding fluorescent signal output [120] (Figure 4c). The authors combined protein hydrolysis-triggered signaling transcriptional events with the trans cleavage activity of the CRISPR/Cas system to achieve dual signal amplification. This strategy was used to detect protease biomarkers at the femtomolar level, with a LOD of 47.8 fM and 5.4 fM for thrombin and matrix metalloproteinase-2 (MMP-2), respectively. The sensitivity of the method was 5–6 orders of magnitude lower than the traditional peptide-based methods. This strategy extends the CRISPR/Cas system to the activity analysis of protease biomarkers, providing a new way for protease activity detection. Given the modularity of PR-Cas, the activity of other proteases can be assessed by simply replacing the PR module.

Furthermore, the CRISPR/Cas system can also be extended for the detection of intermolecular interactions. Kim and coworkers developed a method for the rapid detection of protein/small molecule interactions based on the CRISPR/Cas system using a small molecule modified activator ssDNA (AD) that interacts with the target protein, and the interaction between the small molecule and the protein prevents AD from binding to crRNA, reducing the trans cleavage activity of Cas12a with decreasing fluorescence, which was used for the detection of streptavidin/biotin and antidigoxin/digoxin with the LODs of 0.03 nM and 0.09 nM, respectively, and this process was completed within 11 min (Figure 4d) [121]. In theory, this strategy can be used for the rapid detection of other protein–small molecule interactions, offering a new perspective on protein–small molecule interaction analysis and the screening of related modulators.

## 5. Conclusions and Perspective

CRISPR-based biosensors have achieved huge success in nucleic acid analysis, but studies on the applications of CRISPR for protein detection are still relatively limited. Encouragingly, antibody-combined CRISPR/Cas biosensors have largely improved the LODs of protein detection, and expanded the detection range. It should be noted that most of the CRISPR-based biosensors for protein detection employ aptamers as signal recognition elements because of their superior integration and molecular properties, which easily combine with CRISPR/Cas systems for protein recognition, converting protein signals to nucleic acid signals with activated Cas and signal output. Moreover, with the rapid development of SELEX technology, more and more aptamers for proteins will be discovered [122,123], which will widely expand the applications of the aptamer-assisted CRISPR/Cas biosensors for protein detection.

However, for further routine application and commercialization, the CRISPR/Cas-based protein detection system still faces a range of challenges. The most critical issue is how to efficiently convert the protein signal into a nucleic acid signal, thus activating the trans cleavage activity of Cas enzymes. To date, most of the methods reported so far depend on the antibodies and aptamers, with a few strategies coupled with other methods. These methods generally suffer from problems such as multi-step detection, ease of contamination, the need for specialized technicians, and reduced reliability in real samples. In addition, many CRISPR/Cas-based biosensors for protein detection still need a long detection time and sensitivity that do not fully meet the needs of clinical testing. 

To accelerate the practical application of CRISPR-based biosensors for protein analysis, it will encourage combining CRISPR/Cas systems with other advanced techniques. For example, the emergence of new technologies may better facilitate the development of CRISPR-based biosensing systems for protein detection such as bioinformatics, which could create easy access to predict and design gRNA/crRNA and target activators to improve the sensitivity and specificity of the sensing systems. Automation and high-throughput techniques can be integrated with the CRISPR/Cas system to develop biosensors that can rapidly screen large numbers of samples simultaneously and are easy to perform for protein analysis. Other portable devices (e.g., paper-based devices or microfluidic devices) may also be compatible with CRISPR-based protein sensing systems to meet the needs of clinical analysis. Moreover, as crRNA, PAM sequences, and Mg^2+^ are key to the sensing performance for this type of biosensor, it needs to carefully optimize the reaction system to achieve rapid quantitative protein detection, and improve detection sensitivity and reliability as well as to simplify the detection steps and reduce cost. By combining multiple Cas enzymes with different functions, it may be possible to achieve multiple assays simultaneously. Furthermore, the difficulty in detecting proteins reliably in complicated matrices may be addressed by introducing well-developed preprocessing methods (e.g., extraction, centrifugation, etc.) and encapsulating the components of the sensing system into a restricted space (e.g., DNA technology or liquid–liquid phase separation). Although the developed methods above only have one or two original targets, most of them have the potential to be extended to other proteins by simply changing the aptamer or antibody. Therefore, it is economically efficient to investigate their feasibility in other analytes, which will largely reduce the cost and accelerate their applications. Based on the rapid development of the technology, the deepening research on CRISPR/Cas systems, and the discovery of new CRISPR/Cas systems, we believe that the CRISPR/Cas technology will become one of the mainstream protein detection tools in the future, facilitating its rapid development in disease diagnosis, pathogen analysis, environmental assessment, and other fields.

## Figures and Tables

**Table 1:** A comparison of the antibody-assisted CRISPR/Cas-based strategies for protein detection.

Method	Target	LOD	Detection Range	Signal	Refs.
CRUISE	Cytokine IFN-γ and EGFR	~50 aM	1 fg/mL–1 ng/mL	Fluorescence	[60]
CLISA	IL-6 and VEGF	2.29 fM and 0.81 fM respectively	8 fM–5 pM and 4 fM–2.5 pM, respectively	Fluorescence	[61]
Antibody- DNA barcode conjugated CRISPR/Cas12a	CXCL9	14 pg/mL	-	Fluorescence	[62]
Universal proximity CRISPR/Cas12a	Antibodies	1 pM	-	Fluorescence	[63]
iPCCA	IL-6	100 fM	-	Fluorescence	[63]

## Data Availability

Not applicable.
